# Circulating extracellular vesicle DNA (EV-DNA) and cell-free DNA in ovarian cancer: pathological relevance and diagnostic applications

**DOI:** 10.20517/evcna.2025.120

**Published:** 2026-01-30

**Authors:** Dominic Guanzon, Rakesh Sankar, Pragati Lodha, Reshinthine Purushothaman, Shiyuan Huang, Nanthini Jayabalan, Madushani Dahanayake, Emtiaz Ahmed, Pingping Han, Mostafa Kamal Masud, Yusuke Yamauchi, Carlos Salomon

**Affiliations:** ^1^Translational Extracellular Vesicles in Obstetrics and Gynae-Oncology Group, University of Queensland Centre for Clinical Research, Faculty of Medicine, The University of Queensland, Brisbane QLD 4029, Australia.; ^2^UQ Centre for Extracellular Vesicle nanomedicine, The University of Queensland, Brisbane QLD 4029, Australia.; ^3^Centre for Biomedical Technologies, Queensland University of Technology (QUT), Brisbane QLD 4000, Australia.; ^4^Australian Institute of Bioengineering and Nanotechnology, The University of Queensland, Brisbane QLD 4076, Australia.; ^5^School of Dentistry, Center for Oral-facial Regeneration, Rehabilitation and Reconstruction (COR3), Epigenetics nanodiagnostic and therapeutic group, The University of Queensland, Brisbane QLD 4006, Australia.

**Keywords:** Ovarian cancer, extracellular vesicle-DNA, cell-free DNA, liquid biopsy, nanotechnologies, cancer diagnostics, multi-omics, treatment resistance

## Abstract

Ovarian cancer is one of the leading causes of gynecologic cancer-related mortality in women. However, a significant proportion of ovarian cancer cases are only detected at an advanced stage (III or IV) and are complicated to treat because of metastasis to the peritoneum. This challenge is compounded by vague symptoms and insufficient screening methods for early ovarian cancer detection. A promising solution is liquid biopsy, where the presence of biomarkers (proteins, lipids, and nucleic acids) associated with cancer is identified in the blood circulation. This approach facilitates the real-time monitoring of cancer progression and treatment effects in a non-invasive manner. This contrasts with traditional tumor biopsy, where only a small portion of the tumor is sampled, serial sampling of the tumor is impractical, or sometimes, tumor biopsy is not feasible. This review discusses the cell-free and extracellular vesicle components in blood, highlighting their DNA as a target in liquid biopsies for cancer diagnostics, with a specific emphasis on ovarian cancer. It also underscores the need for further research into the biological underpinnings and functional roles of these DNA fragments to integrate them into multi-omics approaches for detailed insights into tumor biology and treatment resistance in ovarian cancer.

## INTRODUCTION

The National Cancer Institute (USA) states that ovarian cancers are caused by abnormal growth of cells in the ovary, with the most common type being epithelial ovarian cancer^[1]^. Annually, 295,000 women are diagnosed with ovarian cancer worldwide, resulting in a mortality rate of 185,000^[2]^. In February 2024, the Australian government published a report stating that in Australia, approximately 2,000 new cases of ovarian cancer were diagnosed in 2023. An estimated 1 in 87 females, or approximately 1.2% of females, may face the risk of being diagnosed with ovarian cancer by the age of 85 years^[3]^. Accounting for all ovarian cancer stages, the five-year survival rate is only 48%^[4]^. Initially, ovarian cancer was considered as a single disease, but it was later classified into four subtypes based on histological evidence: serous, endometroid, clear cell, and mucinous carcinomas. Among these, the most diagnosed subtype is high-grade serous carcinoma. Unfortunately, ovarian cancers are often diagnosed at an advanced stage (III or IV), thus proving difficult to treat due to the spread of cancer to the peritoneum. This challenge is further exacerbated by non-specific symptoms and a lack of effective screening methods for early detection^[5]^.

A promising solution is liquid biopsy, which identifies biomarkers (proteins, lipids, and nucleic acids) associated with cancer in the blood circulation. This review focuses on circulating DNA molecules, first described by Mandel *et al.* in 1948^[6]^. Among these, circulating cell-free DNA (ccfDNA) is the most well-studied and results from apoptosis or necrosis. Consequently, ccfDNA exhibits a fragmentation pattern of 165-bp increments, reflecting nucleosome organization^[7]^. Biomolecules such as DNA can also be packaged into extracellular vesicles (EVs), which can be found in biological fluids such as blood and are informative of tumor status and chemoresistance^[8,9]^. Interestingly, studies have shown that the fraction of ccfDNA originating from EVs (EV-DNA) is more protected and longer (> 10 kb) than ccfDNA^[10,11]^. In liquid-based diagnostics, circulating DNA can be analyzed for its fragmentation pattern, epigenetic modifications and genetic variations^[12]^. This enables real-time monitoring of cancer progression and treatment effects in a non-invasive manner^[13]^. In contrast, traditional tumor biopsy samples only a small portion of the tumor, making it difficult to capture the complete molecular makeup. Additionally, serial sampling of the tumor is impractical, and sometimes, tumor biopsy is not feasible^[13]^.

The previously described advantages have led to the translation of ccfDNA to clinical practice. One notable example is the use of ccfDNA as a non-invasive prenatal test, which is now available globally^[14]^. This was initially pioneered by Lo *et al.*, who discovered fetal ccfDNA in maternal circulation for the detection of trisomy 21 in pregnant women^[15,16]^. In the context of oncology, the Galleri Test developed by Grail is a ccfDNA methylation-based multi-cancer early detection assay^[17]^. This test is under rigorous evaluation in clinical trials across diverse populations, assessing its safety and integration into routine screening programs^[18]^. In contrast, EV-DNA is still in early development and faces several barriers to clinical implementation in health care systems. These challenges include standardization of isolation methods to ensure purity and yield, as well as enhancing downstream detection methods to improve sensitivity and specificity^[19]^. However, EV-DNA has unique advantages compared to ccfDNA, such as greater protection and longer length^[10,11]^. Thus, overcoming these challenges could unlock the potential of EV-DNA as a biomarker for future diagnostic applications, especially as new detection technologies emerge.

In routine pathology, a range of conventional detection methods can be used to analyze this ccfDNA, such as real-time quantitative polymerase chain reaction (PCR), digital PCR, and short-read next-generation sequencing (NGS)^[20]^. While quantitative PCR (qPCR) is widely used and is cost-effective, digital PCR offers absolute concentration and high sensitivity, critical for detecting rare genetic variants. However, PCR requires prior knowledge of the target sequence, whereas NGS is more comprehensive^[20]^. New emerging technologies are also advancing the analysis of ccfDNA and EV-DNA in diagnostics. For example, Oxford Nanopore Technologies (ONT) has developed a long-read sequencer^[12]^, which can leverage the long EV-DNA properties to enable greater diagnostic utility and accuracy. This is achieved through long-read sequencing techniques such as genome phasing, which separates genetic mutations into maternal/paternal origins^[21]^. Further advances in nanomaterial-based technologies and high-sensitivity sensors show promise in improving EV isolation methods and analysis of their biomolecules, including DNA^[22]^.

In this review, we will summarize recent advances in liquid biopsy technologies and the clinical applications of circulating DNA, in particular ccfDNA and EV-DNA, in oncology, including their relevance to ovarian cancer. We will discuss the biological characteristics of these DNA molecules, their isolation and analysis methodologies, and their diagnostic potential. In addition, we will cover new technologies - such as long-read sequencing and nanomaterials-based techniques - that are enhancing the sensitivity and specificity of liquid biopsy tests. Finally, we will discuss the current challenges and achievements in translating ccfDNA and EV-DNA into clinical practice.

## EV-DNA

EVs are membrane-bound vesicles released by cells into biological fluids or extracellular spaces. They can be classified based on their size and cellular origin, with small EVs (< 200 nm) being the most widely studied^[23]^. Furthermore, small EVs contain and protect molecular cargo, such as nucleic acids, proteins, lipids, and metabolites. These EVs mediate intercellular communication by delivering their molecular cargo from donors to recipient cells. Consequently, EVs are involved in a range of normal and pathological processes such as ovarian cancer progression, recurrence and chemoresistance^[8,9]^. Measuring EV molecular cargo can thus be used as a tool for ovarian cancer diagnostics; though previous studies have predominantly focused on RNA and proteins, we focused on the relatively unexplored domain of EV-DNA in this review.

### EV-DNA properties and release mechanism from cells

The DNA content *in vitro* is reported to be higher in EVs released by tumor cells than in those released by normal cells^[24]^. Interestingly, EV subpopulations harbor distinct double-stranded genomic DNA cargos that carry cancer-associated mutations^[25]^. In addition to double-stranded genomic DNA^[25-28]^, EVs contain single-stranded DNA (ssDNA)^[29]^, extrachromosomal DNA^[30]^, and mitochondrial DNA (mDNA)^[27,28,31-33]^. Currently, there is no consensus on the specific location of EV-DNA. Research indicates that its presence is confined to the inside of EVs^[34]^, while some studies suggest it is external^[27]^, and other experimental findings indicate both internal and external locations^[35,36]^. Furthermore, the length of EVs ranges from > 10 kb in small serum EVs from patients with pancreatic cancer^[10]^ to 2 million base pairs in large plasma EVs from patients with prostate cancer^[29]^. In an interesting study that analyzed single EVs using nanoflow cytometry, the length of EV-DNA was reported to range from 200 base pairs to 550 kilobase pairs^[36]^.

Packaging of DNA into EVs, such as exosomes, plays an important role in cellular homeostasis by removing harmful cytoplasmic DNA originating from the nucleus^[37]^. However, the exact mechanisms underlying DNA packaging into EVs remain poorly understood. A study by Reis-Sobreiro *et al.* revealed that low levels of emerin cause nuclear shape instability and membrane blebbing, leading to the formation of large vesicles that can be shed from cells^[38]^. Another study indicated that micronuclei with CD63 surrounding the nuclear envelope are responsible for loading nuclear DNA into exosomes^[35]^. Immunoprecipitation experiments have revealed that CD63 forms a complex with histone H2B and genomic DNA, which may facilitate DNA loading into exosomes^[35]^. A separate study reported that tumor and intestinal epithelial cells release host DNA in an exosome-dependent manner in response to chemotherapy drugs^[39]^. In contrast, a comprehensive profile of exosome composition indicated that they are not vehicles of active DNA release^[40]^.

In summary, EVs carry diverse DNA cargoes, including genomic DNA and mDNA, with variations in length across different cancer types. The exact location (internal, external, or both) and whether the single- or double-stranded nature of EV-DNA remains uncertain. Although the presence of various forms of EV-DNA has been highlighted, the precise mechanisms governing their encapsulation remain elusive. Mechanisms such as membrane blebbing, micronuclei formation, and protein-DNA interactions involving CD63 and histone H2B have been proposed. Further, the possibility that DNA may not be encapsulated in EVs adds another layer of complexity. These discrepancies between studies may be attributed to the diverse methodologies employed for EV isolation and the inherent heterogeneity of EV subpopulations.

### Isolation techniques and analytical approaches for cancer detection

Various commercial kits are used to isolate DNA from EVs, including the QIAamp DNA mini kit (Qiagen, Hilden, Germany)^[27,36,41-43]^, QIAamp DNA micro kit (Qiagen)^[11,42,44-46]^, and QIAamp circulating nucleic acid kit (Qiagen)^[47,48]^; DNeasy blood and tissue kit (Qiagen)^[29]^; XCF Exosomal DNA isolation kit (SBI)^[42,49]^; SeleCTEV exosomal kit (Exosomics)^[42]^; Genomic DNA mini kit (Geneaid)^[50]^; and Maxwell® RSC ccfDNA Plasma Kit (Promega)^[51]^. Studies have also demonstrated the use of the traditional phenol-chloroform-isoamyl alcohol extraction^[42,52]^ or QIAzol Lysis Reagent (Qiagen)^[53]^ for EV-DNA isolation. Overall, a wide array of commercially available kits and traditional methods are employed for DNA isolation from EVs [Table 1], reflecting the diverse approaches in the field. However, the variability in methodologies, coupled with the different isolation techniques and sources of EVs, poses a challenge for directly comparing results across studies.

**Table 1:** Kits used for EV-DNA isolation

**DNA extraction kit**	**EV source**	**EV isolation method**	**Ref.**
QIAamp DNA mini kit (Qiagen)	• Cell culture media • Serum	• Differential ultracentrifugation • Precipitation • Differential ultracentrifugation followed by iodixanol density gradient separation or sucrose cushion	[27,36,41-43]
QIAamp DNA micro kit (Qiagen)	• Plasma • Cell culture media	• Differential ultracentrifugation • Differential ultracentrifugation followed by a sucrose cushion • Size exclusion chromatography	[11,42,44-46]
QIAamp circulating nucleic acid kit (Qiagen)	• Plasma	• Differential ultracentrifugation	[47,48]
DNeasy blood and tissue kit (Qiagen)	• Cell culture media	• Differential ultracentrifugation followed by iodixanol density gradient separation	[29]
XCF exosomal DNA isolation kit (SBI)	• Cell culture media • Plasma	• Differential ultracentrifugation • Size exclusion chromatography • Differential ultracentrifugation followed by a sucrose cushion	[42,49]
SeleCTEV exosomal kit (Exosomics)	• Plasma	• Differential ultracentrifugation followed by a sucrose cushion	[42]
Genomic DNA mini kit (Geneaid)	• Cell culture media	• Differential ultracentrifugation followed by iodixanol density gradient separation	[50]
Maxwell® RSC ccfDNA Plasma Kit	• Human plasma	• Differential ultracentrifugation	[51]
QIAzol/phenol-chloroform-isoamyl alcohol	• Plasma	• Membrane affinity spin columns • Differential ultracentrifugation • Differential ultracentrifugation followed by sucrose cushion	[42,52,53]

EV: Extracellular vesicle.

Various techniques, such as PCR, microarrays, and NGS, have been used to analyze EV-DNA and to reveal variants/alterations in DNA sequences or epigenetic modifications, such as methylation [Table 2]. For example, targeted NGS based on capture has been used to identify clinically actionable EV-DNA mutations in lung adenocarcinoma, demonstrating high concordance with matched cancer tissues^[54]^. Furthermore, a study using whole-genome and targeted NGS found that nuclear DNA and EV-derived mDNA (EV-mDNA) were longer than cell-free plasma DNA^[46]^. Additionally, EV-mDNA copy numbers were decreased in patients with hepatocellular carcinoma. Through whole-genome sequencing, microarrays, and allele-specific PCR, EV-DNA has been determined to cover the entire genome without bias, accurately reflecting the mutation status of parental melanoma cell lines^[26]^. Whole-genome sequencing of EV-DNA revealed copy number variations commonly associated with prostate cancer that reflected the cells of origin^[29]^. In contrast, whole-genome sequencing has revealed that EV-DNA copy number alterations or variants are restricted to specific genomic loci in patients with breast and metastatic cancers^[45,47]^.

**Table 2:** Methods to analyze EV-DNA methylation and genetic variants in cancer

**EV source**	**EV isolation method**	**Variant detection method**	**Methylation detection method**	**Ref.**
Glioblastoma stem-like cell cells	• Differential ultracentrifugation • Size exclusion chromatography	• Targeted NGS • Digital PCR	• Infinium methylation EPIC arrays	[49]
Murine melanoma cells (B16-F10)	• Differential ultracentrifugation	• Whole genome sequencing • Microarray • Allele-specific PCR	• Methylation-specific antibodies with dot blot readout	[26]
Human plasma from prostate cancer patients	• Membrane affinity spin columns		• Bisulfite MSP	[53]
Human urine from prostate cancer patients	• Differential ultracentrifugation • Differential ultracentrifugation and precipitation		• Reduced representation bisulfite sequencing • Digital PCR	[43]
Human bronchoalveolar lavage	• Differential ultracentrifugation	• Capture-based targeted NGS		[54]
Human plasma	• Differential ultracentrifugation	• Whole genome sequencing • Capture-based targeted NGS		[45-47]
Human plasma	• Differential ultracentrifugation	• Digital PCR		[48,51,52]
Human plasma and prostate cancer cell lines (PC-3)	• Differential ultracentrifugation • Differential ultracentrifugation followed by iodixanol density gradient separation	• Whole genome sequencing • Digital PCR		[29]

EV: Extracellular vesicle; NGS: next-generation sequencing; PCR: polymerase chain reaction; MSP: methylation-specific PCR.

Nevertheless, the limited amount of DNA found within EVs presents an analytical hurdle^[55]^, prompting researchers to explore methodologies such as digital PCR, which offer higher sensitivity than NGS^[56]^. For example, consistent monitoring of EV-DNA Kirsten rat sarcoma viral oncogene homolog (*KRAS*) mutations by digital PCR in individuals with pancreatic cancer can provide insights into the effectiveness of neoadjuvant chemotherapy and disease progression^[48]^. Similarly, a separate study using digital PCR demonstrated the potential of EV-DNA to monitor *KRAS* and B-Raf proto-oncogene, serine/threonine kinase (*BRAF*) mutations in patients’ blood with colorectal cancer^[51]^. Additionally, another study measuring mutant *KRAS* EV-DNA by digital PCR found it elevated in early- and late-stage pancreatic ductal adenocarcinoma^[52]^. Elevated MYC proto-oncogene (*MYC*)/phosphatase and tensin homolog (*PTEN*) EV-DNA ratios, detected by digital PCR, have also been reported in patients with prostate cancer^[29]^. Finally, microarray and digital PCR techniques demonstrated that EV-DNA mutations and copy number variations mirrored those of the original glioblastoma^[49]^.

In addition to evaluating cancer-specific mutations in EV-DNA, researchers have examined epigenetic alterations, such as DNA methylation. An effective method for examining overall EV-DNA methylation involves antibodies targeting 5-methylcytosine. This method revealed DNA methylation profiles in EVs that closely mirrored those found in the original murine melanoma cell lines^[26]^. Sensitive techniques involving methylation-specific PCR (MSP) have also revealed a correlation between the methylation of glutathione S-transferase P1 (*GSTP1*) and Ras ASSociation domain Family 1A (*RASSF1A*) genes in EV-DNA and reduced overall survival in patients with prostate cancer^[53]^. Employing comprehensive methylation profiling techniques, such as Infinium Methylation EPIC arrays, on EV-DNA derived from glioma samples accurately reflects the genome-wide methylation profile of the original glioblastomas, regardless of the EV isolation technique used^[49]^. Finally, global (reduced-representation bisulfite sequencing) and targeted (methylation-specific digital PCR) techniques applied to EV-DNA extracted from urine revealed that the methylation status of *RASSF1A* could distinguish patients with prostate cancer from controls^[43]^.

These studies have employed traditional methods to examine EV-DNA as a biomarker for cancer diagnosis and prognosis prediction by identifying mutations and epigenetic changes. Nonetheless, given the long nature of EV-DNA^[10,29,36]^, leveraging advanced long-read sequencing technologies such as Oxford Nanopore offers an opportunity to gain a more comprehensive understanding of genomic alterations and methylation patterns. This is facilitated by the ability to capture more information from the same DNA strand, enabling simultaneous analysis of genomic alterations, 5-methylcytosine, 5-hydroxymethylcytosine, and 6-methyladenosine within the same dataset^[57]^, and is less prone to guanine-cytosine (GC) bias^[58]^. In contrast, traditional methods often require separate laboratory conversion techniques, such as bisulfite conversion, to analyze these variations^[43]^. Several studies have used long-read ONT sequencing to explore the role of ccfDNA in cancer pathology. These studies explored various aspects of ccfDNA, including methylation patterns, nucleosome positioning, copy number alterations, and fragmentation^[12,59,60]^. Notably, tumor signals have been detected in long ccfDNAs^[60]^. As EV-DNA comprises a portion of ccfDNA (which represents the entirety of the lysed material), some of the long ccfDNA may be considered to originate from EVs (i.e., EV-DNA). Although long-read sequencing has been applied to ccfDNA, studies focusing on EV-DNA using this method are scarce, particularly in the context of ovarian cancer. Consequently, the clear benefits of long-read sequencing coupled with long EV-DNA may offer valuable insights into ovarian cancer pathology and treatment strategies.

### EV-DNA in ovarian cancer

A recent study conducted a comprehensive nucleic acid analysis of circulating EVs in patients with high-grade serous ovarian cancer to identify biomarkers of treatment response^[61]^. They targeted patients with no residual disease after primary surgery, those with excellent responses to neoadjuvant chemotherapy, and those with poor responses to neoadjuvant chemotherapy. Whole-genome sequencing of EV-DNA revealed alterations in pathways such as receptor tyrosine kinase (RTK)-RAS and Wnt, as well as tumor protein p53 (*TP53*) gene mutations^[61]^; the latter was also examined in spheroids and ccfDNA from ovarian cancer ascites^[62]^. Furthermore, EV-DNA gene mutations in 38% of the patients were associated with ovarian cancer, whereas 92% of mutations were in hallmark cancer genes^[61]^. Despite their potential as liquid biopsy tools for cancer detection, these EV-DNA mutations did not show differential expression in response to chemotherapy. Similarly, extracellular vesicle RNA (EV-RNA) could not distinguish chemotherapy responses but could differentiate between patients with no residual disease after surgery and those undergoing neoadjuvant chemotherapy. Interestingly, the EV-DNA genome and EV-RNA transcriptome did not reflect the tissue, but provided insights into the complex genomic landscape of the tumor microenvironment^[61]^.

In contrast, the copy number variation in the DNA between ovarian cancer cell line OVCAR-3 and its EVs was quite similar when examined *in vitro*^[35]^. This similarity extends to *in vivo* observations, where EV-DNA extracted from ascites mirrors the tumor, but not when extracted from plasma. Furthermore, CD63 knockdown in OVCAR-5 cells reduced the quantity of EVs carrying nuclear DNA, suggesting that CD63 plays an important role in DNA packaging within EVs in ovarian cancer^[35]^. Consistent with a previous study, ultracentrifugation (UC) of ascitic fluid from women with ovarian cancer revealed the presence of high-molecular-weight EV-DNA, whereas the supernatant showed a pattern of ccfDNA (150-base-pair increments)^[62]^. The association of this high-molecular-weight DNA with EVs makes it particularly advantageous for genomic sequencing techniques that rely on long reads. In addition to EV genomic DNA, EV-mDNA has a higher copy number in plasma-derived EVs from patients with late-stage ovarian cancer than in those from healthy controls^[63]^. Notably, cell-free mDNA cannot perform the same, highlighting the advantages of using EV-mDNA in ovarian cancer diagnostics.

Similar to EV-mDNA, ovarian cancer cell lines (A2780) resistant to the chemotherapy drug cisplatin exhibited elevated EV-mDNA levels and released higher quantities of EVs^[64]^. A similar increase in nuclear EV-DNA was observed in OVCAR-5 cells exposed to genotoxic drugs^[35]^. Interestingly, EVs derived from cisplatin-resistant ovarian cancer cells confer a chemoresistant phenotype in cisplatin-sensitive ovarian cancer cells^[64]^. However, the specific role of EV-DNA in acquired chemoresistance remains unclear. Some insight can be gained from another study focusing on colorectal cancer cell lines, which found that the chemotherapy drug irinotecan increased the release of double-stranded DNA via EVs. This DNA uptake by phagocytes led to inflammation, characterized by elevated interleukin (IL)-1β and IL-18 levels^[39]^. Chronic inflammation induced by therapy, including IL-1β, is known to significantly contribute to treatment resistance and cancer progression^[65]^. The chemoresistance acquired in ovarian cancer cells reported earlier^[64]^ may be attributed to the delivery of EV-DNA, which triggers inflammation and subsequent chemoresistance.

### Conventional diagnostic methods for EV-DNA

The isolation and detection of bodily fluid-derived EVs encounter challenges owing to their low concentration in circulation and the presence of abundant impurities with similar properties. Traditional isolation techniques, including UC, density gradient centrifugation (DGC), polymer-based precipitation, and size-exclusion chromatography (SEC), primarily rely on physical properties such as size, density, and solubility^[66]^.

UC, which separates particles based on their sedimentation coefficients, is the most common method for EV isolation. It precipitates EVs at high speed (~100,000 × *g*) while removing large debris and unwanted particles at low speeds. Other factors, such as input volume, sample type, number of centrifugation cycles, and rotor type, should also be optimized, as they affect the purity and yield of the resulting solution^[67]^. Furthermore, the DGC method allows particles to reach a balance between centrifugal force and resistance in a density gradient solution^[68]^. It can yield higher purity than UC but requires an extensive amount of time, biofluid volume, and experimental proficiency. Similarly, polymer-based precipitation is a popular approach for low-input EV isolation but has been reported to cause severe contamination^[69]^. Although SEC can separate particles of different sizes based on the molecular sieve effect, its purity remains lower than that of conventional UC and DGC^[70]^. To improve efficiency and quality, combining multiple methods, such as UC with DGC, can be beneficial. Combining SEC with UC/DGC can also improve purity^[71,72]^.

Traditional EV detection methods are generally categorized into quantification, visualization, and biochemical measurements^[66]^. Quantification techniques include nanoparticle (NP) tracking analysis, dynamic light scattering, and tunable resistive pulse sensing, whose performance largely depends on the efficiency and purity of the isolation approaches^[73]^. Visualization techniques include scanning electron microscopy, transmission electron microscopy, and cryo-electron microscopy, but artificial perturbations from sample-handling procedures can affect these results^[74]^. Biochemical assays, such as western blotting, PCR, and enzyme-linked immunosorbent assay (ELISA), are highly specialized but are hindered by the heterogeneity of EVs, which can lead to nonspecific signals that obscure disease-specific biomarkers^[75]^. Clinical factors such as age and sex further complicate detection and analysis. Although guidelines such as Minimal Information for Studies of Extracellular Vesicles 2018 (M ISEV2018) have improved the standardization of EV research, the complexity of evolving technologies continues to introduce new challenges^[76,77]^.

### Clinical translation of EV-DNA

The most prominent challenge in ovarian cancer diagnosis is the short window of detection because early diagnosis is crucial for improving disease outcomes and enabling timely therapeutic interventions^[78]^. Current diagnostic tools, such as biomarker tests and imaging, often lack the sensitivity and specificity required for early disease detection, which is essential for improving survival rates. Biomarkers such as Cancer Antigen-125 (CA-125)^[79]^, human epididymis secretory protein^[80]^, and osteopontin^[81]^ are commonly used in combination with imaging techniques.

However, a significant limitation of these biomarkers is that their levels can also increase under non-cancerous conditions. For example, CA-125 is elevated in conditions such as endometriosis and benign ovarian cysts, as well as in premenopausal women, leading to the false-positive detection of ovarian cancer^[82,83]^. Furthermore, many ovarian cancers, especially in the early stages, do not cause substantial changes in biomarker levels, making disease detection difficult until it reaches a more advanced and less treatable stage^[84]^. This lack of specificity and sensitivity reduces the effectiveness of current biomarker-based diagnostics, underscoring the need for more accurate, reliable, and early detection methods for ovarian cancer.

EVs have emerged as promising biomarkers for ovarian cancer because of their ability to carry tumor-derived genetic material, proteins, and lipids that reflect tumor molecular characteristics^[85]^. These vesicles serve as carriers of key biological molecules from the tumor microenvironment, providing a snapshot of the tumor’s genetic and molecular profile^[86]^. Among EV-associated genetic materials, microRNAs (miRNAs) have attracted significant attention for their ability to provide crucial insights into tumor biology^[87]^.

Although much of the focus has been on EV-associated proteins and RNA biomarkers, DNA within EVs has largely been overlooked, despite its growing potential. However, EV-DNA has emerged as a crucial player in various diseases, significantly expanding the diagnostic and therapeutic possibilities of EVs. EV-DNA reflects both nuclear and mitochondrial genomes, making it particularly valuable for understanding the pathophysiology of diseases such as ovarian cancer^[63]^. This DNA can provide essential information regarding genetic mutations, epigenetic changes, and tumor evolution, offering a more comprehensive understanding of the disease.

The potential use of EV-DNA as a diagnostic and prognostic biomarker has been demonstrated under various pathophysiological conditions, highlighting its broad applicability for disease detection and monitoring. Several studies have shown the utility of EV-DNA as a diagnostic biomarker in neurodegenerative diseases^[32,88]^, such as Parkinson’s, where damaged mDNA within EVs plays a crucial role in disease progression, offering a novel target for diagnosis and monitoring^[88]^. In periodontitis diagnosis, global 5-methylcytosine DNA methylation and hypermethylation of promoter regions in inflammatory genes, such as tumor necrosis factor-α (*TNF-α*), were linked to periodontitis pathogenesis compared with periodontally healthy individuals^[89-93]^. In cardiovascular diseases (CVD), a global health burden, mDNA fluctuations have been reported, with one prospective case-control study demonstrating higher EV-mDNA levels in patients with CVD, but no differences in buffy coat or cell-free DNA (cfDNA)^[94]^. This strongly supports the potential of EV-mDNA as a biomarker for CVD diagnosis. In cancer, EV-DNA shows considerable promise as a diagnostic biomarker reflecting the genetic alterations in primary tumors. For instance, in patients with stage 3 melanoma, EVs derived from seromas show BRAF^V600E^ mutations, which correlated with a higher risk of relapse^[95]^. This demonstrates the potential of EV-DNA for cancer detection and for personalized treatment strategies.

EV-DNA holds great promise as a cancer diagnostic tool. However, its use for cancer diagnosis is still in preliminary stages and faces several challenges that need to be addressed. First, EV isolation from biological fluids must be standardized to ensure the purity^[96]^ and reproducibility of EV-DNA for diagnostic applications. Inconsistent isolation techniques can lead to contaminated samples with co-isolation of other particles and proteins^[97]^, which can affect the accuracy of subsequent analyses and hinder the reliability of the results. Second, the integrity of EV-DNA can vary depending on factors such as the source of EVs and storage conditions, potentially impacting its clinical utility. DNA in EVs is susceptible to degradation over time, compromising its ability to serve as a reliable biomarker. Therefore, establishing and implementing global regulations and standardized protocols is essential for sourcing, handling, storing, and isolating EVs^[98]^. This will ensure consistency across laboratories and countries, ultimately enhancing the reliability and reproducibility of EV-DNA biomarker studies^[99,100]^. Addressing these challenges is critical for advancing the use of EV-DNA as a cancer diagnostic tool.

## ccfDNA

The term “circulating” refers to all extracellular molecules released into peripheral blood, including nucleic acids such as nuclear DNA or mDNA^[101]^. The presence of nucleic acids in the blood of both healthy and diseased patients was first demonstrated in 1948^[6]^. Later, in 1989, a fraction of this circulating DNA was found to originate from tumor cells^[102]^. Subsequent studies revealed that these circulating DNAs also contain mutations that may be useful for cancer detection and monitoring^[103,104]^. At this time, the idea of a “liquid biopsy” emerged, based on the diagnostic use of genetic alterations in circulating nucleic acids. A more comprehensive historical overview and function of circulating DNA can be found in the work by Thierry *et al.*^[101]^. Currently, one of the most well-studied forms of circulating nucleic acid is ccfDNA, which refers to the DNA fragments released into blood during apoptosis or necrosis^[105]^. Among the earliest and most significant applications of ccfDNA is non-invasive prenatal screening. The discovery of fetal ccfDNA in maternal circulation was pioneered by Lo *et al.*^[15,16]^, initially for the detection of trisomy 21. A subsequent large validation study reported exceptional performance, with sensitivities as high as 100% and specificities of approximately 98%^[90]^. Since its clinical introduction in 2011, this technology has been applied to more than 700,000 plasma samples from over 50 countries within a three-year period, underscoring its rapid adoption and impact on prenatal care^[15,16]^. The following sections detail the specific properties and applications of ccfDNA, highlighting its emerging role in oncology and ovarian cancer.

### ccfDNA properties and release mechanism from cells

During apoptosis or necrosis, DNA fragments are released into the bloodstream as ccfDNA, and a fraction of these, in patients with cancer, are derived from tumor cells undergoing these processes^[105]^. Typically, ccfDNA molecules are double-stranded at approximately 165 bp increments, a fragmentation pattern reflecting nucleosome organization^[7]^. However, DNA fragments greater than 10,000 base pairs have also been reported^[106]^. These passive processes are often enhanced in various pathological conditions, including cancer, trauma, and inflammatory diseases, in which higher rates of cell turnover and tissue damage result in increased levels of detectable ccfDNA. For example, in healthy individuals, the ccfDNA concentration in plasma can range from 1 to 10 ng/mL^[107]^. In contrast, patients with cancer have higher levels, averaging 13 ng/mL of plasma for cancer stages I to III^[107]^, or ranging from 0.5 ng/mL to 1.1 ug/mL of plasma in a cancer cohort consisting of 21 tumor types^[108]^. Although the main mechanism of ccfDNA release is thought to be via apoptosis, a passive process, several reports have described active mechanisms for its release^[109,110]^, including its secretion through EVs, a concept already discussed in this review.

### ccfDNA in ovarian cancer

Growing evidence supports the use of ccfDNA-based assays for cancer screening, minimal residual disease detection, treatment response evaluation, and real-time assessment of resistance mechanisms^[111]^. Among various molecular alterations observed in ccfDNA, including point mutations, the most advantageous is DNA methylation. One reason is that DNA methylation changes occur early in tumorigenesis and are stable^[112]^. Consequently, numerous studies have explored the potential of ccfDNA methylation assays in ovarian cancer detection. One such assay, OvaPrint, was designed to distinguish high-grade serous ovarian carcinomas from benign masses^[113]^. The assay demonstrated an overall accuracy of 91%, a positive predictive value of 0.95, and a negative predictive value of 0.88, which is better than those of other commercially available tests.

To further reinforce the role of methylation markers, differentially methylated regions identified in ccfDNA were used to discriminate tumors from non-tumor groups, with high sensitivity and specificity, showing receiver operating characteristic values ranging from 0.86 to 0.98^[114]^. A meta-analysis of 18 studies confirmed these findings, with an overall strong diagnostic accuracy (median 85%, range 40%-91%) of serum/plasma ccfDNA methylation tests^[112]^. This accuracy improves when multiple genes are tested and when malignant lesions are compared with benign pelvic masses. Notably, *RASSF1A* was the most frequently investigated gene, analyzed in eight of these studies^[112]^.

Beyond methylation, several novel ccfDNA-based approaches exploit different genomic and epigenomic characteristics to enhance diagnostic performance. For example, Zhou *et al.* used low-pass whole-genome sequencing of ccfDNA to integrate copy number variation, 5′-end motifs, fragmentation patterns, and nucleosome footprinting into a composite ovarian cancer score^[115]^. This score achieved an area under the curve of 97.7% with 94.7% sensitivity and 98.0% specificity for distinguishing patients with ovarian cancer from healthy controls. Furthermore, high levels of ccfDNA have been reported as independent predictors of disease-specific survival and have been shown to be superior to CA125 in predicting mortality, underscoring the prognostic value of ccfDNA in ovarian cancer^[116]^. All the studies described above highlight that ccfDNA may be a multifunctional biomarker with the potential to improve early detection, guide therapeutic decisions, and monitor disease courses.

### Conventional diagnostic methods for ccfDNA

Although the detection and analysis of ccfDNA holds great promise for disease diagnostics, several challenges complicate this potential. These challenges include the very low concentrations of ccfDNA in biological samples, their fragmentation, and inherent biological variability, all of which affect sensitivity and interpretation^[20,117]^. Despite these challenges, several traditional diagnostic methods have been developed over the past few decades, offering noninvasive approaches for the isolation, detection, and analysis of ccfDNA.

The isolation of ccfDNA from complex biological samples is a critical first step in ccfDNA-based research. Given the low concentrations and fragmented nature of ccfDNA, obtaining high-quality, intact DNA is challenging but crucial for downstream applications, including diagnostics and genetic analysis. Several kits are commercially available for the extraction and isolation of ccfDNA from blood plasma and serum such as the QIAamp DNA Blood Mini Kit^[118]^, Phenol-chloroform method^[119]^, Qiagen’s QIAamp circulating nucleic acid kit, Phenol-chloroform method^[119]^, Qiagen’s QIAamp circulating nucleic acid kit^[120]^, MagMAX™ Cell-Free DNA Isolation Kit^[121]^, PHASIFY™ (Phase Scientific International Limited, Kwun Tong, Hong Kong, China)^[121]^, NucleoSpin® Gel and PCR Clean-up (Macherey-Nagel), and NucleoSpin® Plasma XS (Macherey-Nagel)^[122]^. The ccfDNA concentration can be measured using the Quantum Bit (QUBIT) assay, and Ultraviolet/Nanodrop (UV/Nanodrop) spectroscopy is used to determine DNA purity^[123]^. After isolation, different conventional methods are used for the downstream analysis of ccfDNA, as discussed below.

One of the earliest and most widely adopted methods for ccfDNA analysis is real-time qPCR, which amplifies specific DNA sequences. qPCR is particularly useful for detecting mutations in cancer-related genes and has been extensively employed to monitor patients with cancer^[124,125]^. However, it requires prior knowledge of target mutations or sequences, limiting its utility for broader mutation profiling. Despite this limitation, qPCR remains a fundamental tool for ccfDNA analysis because of its accuracy and comparatively shorter turnaround time^[20]^. Digital droplet PCR (ddPCR) is another conventional method used to detect and quantify ccfDNA. In this approach, a DNA sample is partitioned into thousands of droplets, each undergoing independent PCR amplification. As a result, ddPCR is particularly advantageous for detecting low-frequency mutations, such as those found in early-stage cancers or minimal residual disease after treatment. This high precision makes ddPCR a valuable tool for monitoring disease progression and treatment responses in patients with cancer^[126,127]^. However, similar to qPCR, ddPCR requires prior knowledge of the mutation or genomic region of interest. MSP is another important technique used in ccfDNA analysis, particularly for cancer detection. Aberrant DNA methylation patterns are a hallmark of cancer and MSP enables the detection of these epigenetic modifications in ccfDNA. This method has been successfully applied to detect tumor-specific methylation patterns in ccfDNA, providing a noninvasive approach to cancer diagnosis^[128,129]^.

Another major approach is NGS, which enables comprehensive analysis of ccfDNA, including whole-genome sequencing or targeted sequencing of specific genomic regions. NGS offers unparalleled coverage and the ability to detect a wide range of genetic alterations, including single-nucleotide variants, insertions and deletions, copy-number variations, and epigenetic changes. This capability makes NGS a highly versatile tool for cancer diagnosis, enabling the identification of previously unknown mutations^[124,130,131]^. For example, using long-read ONT sequencing, tumor burden can be monitored in patients with cancer by measuring tumor-specific ccfDNA quantity and methylation^[59]^. Another study on ccfDNA showed that long-read ONT sequencing can distinguish cancer cases from controls by examining methylation, nucleosome positioning, copy number alterations, and fragmentation^[12]^. Similarly, copy number alterations, nucleosome positioning, and fragment analysis of ccfDNA from patients with lung and bladder cancers have been performed using sequencing^[60]^. Despite these advantages, NGS is expensive, requires more input DNA than PCR-based methods, and has longer processing times, which can limit its routine clinical application^[132]^. Nevertheless, the broad applicability and depth of analysis offered by NGS make it a key tool in the future of personalized medicine.

Mass spectrometry (MS) is another powerful analytical technique that can be used to analyze ccfDNA and provide insights into its composition and structural features. MS involves ionizing ccfDNA and measuring the mass-to-charge ratios of the resulting ions. This allows detection of specific DNA sequences and modifications (such as methylation), which can provide information about the biological origin of ccfDNA (e.g., tumor-derived vs. non-tumor-derived)^[133,134]^. Traditional methods, such as capillary electrophoresis using the Agilent 2100 Bioanalyzer, can separate DNA fragments based on their size and charge, enabling detailed characterization of ccfDNA fragment size distributions. By examining fragment size distributions, researchers can obtain information about the fragmentation patterns associated with various diseases, including the presence of tumors^[135,136]^.

Although these traditional methods have significantly advanced ccfDNA analysis, they have some limitations. Techniques such as qPCR, ddPCR, MSP, and capillary electrophoresis are limited in their detection range and often require prior knowledge of mutations. Further, although NGS and MS are comprehensive, they are expensive and may not be feasible in all clinical settings. Additionally, the low concentration of ccfDNA in plasma poses challenges for all methods, requiring highly sensitive technologies for accurate detection.

### Clinical translation of ccfDNA

ccfDNA has gained recognition as a valuable biomarker with significant potential for clinical applications in various medical fields. Beyond its initial applications, ccfDNA analysis has emerged as a minimally invasive tool for cancer detection and clinical monitoring in oncology. Medina *et al.* developed a strategy for combining ccfDNA fragmentome analysis with protein biomarker profiling in ovarian cancer^[137]^. Their strategy was achieved using a machine learning framework with > 99% specificity and stage-dependent sensitivities of 72%, 69%, 87%, and 100% for stages I-IV, respectively. Notably, this significantly outperformed the clinically used CA-125, which showed drastically lower sensitivities in the early stages, as low as 34% in stage I^[137]^. Using a similar approach, this research group previously achieved success in clinical trials, leading to the development of the FirstLook ccfDNA blood test for lung cancer using DNA Evaluation of Fragments for Early Interception (DELFI) Diagnostics^[138]^. This highlights the promising potential of ovarian cancer testing for future clinical applications.

Several ccfDNA assays have been used for clinical applications in non-small cell lung cancer. For example, the Guardant360 NGS assay by Guardant Health demonstrated detection rates of non-small cell lung cancer-associated ccfDNA biomarkers comparable to the standard of care tissue genotyping^[139]^. Importantly, combining tissue- and plasma-based genotyping increased the overall frequency of detected driver mutations, while plasma-based testing reduced the median turnaround time from 15 to 9 days. The InVisionFirst Lung Assay by NeoGenomics showed excellent concordance with tissue profiling in a multicenter prospective study^[140]^. This assay not only met the sensitivity and specificity requirements of Food and Drug Administration (FDA)-approved single-gene circulating tumor DNA (ctDNA) assays but also identified 26% more actionable alterations than those with conventional tissue testing.

The Foundation One Liquid Companion Diagnostic (CDx) from Foundation Medicine represents a milestone in pan-cancer genomic profiling and extends the clinical use of ccfDNA diagnostics. It is the first tissue-based companion diagnostic approved by the Food and Drug Administration through both analytical and clinical validation across all solid tumors^[141]^. The robust performance in detecting genomic alterations predictive of the response to targeted therapies underscores the potential of this assay to guide precision oncology. Another application of ccfDNA is the Galleri Test, a methylation-based multi-cancer early detection assay developed by Grail^[17]^. For a more in-depth discussion on ccfDNA-based assays in clinical practice, readers may refer to the work of García-Pardo *et al.*^[142]^. The performance of the Galleri Test is under rigorous evaluation in clinical trials across diverse populations, assessing its safety profiles and determining its integration into routine screening programs^[18]^. These studies are critical to establish the clinical utility of the Galleri Test and other multi-cancer early detection approaches in reducing cancer-related morbidity and mortality.

## NANOTECHNOLOGIES IN EV RESEARCH

Nanomaterial-driven nanotechnologies have significantly improved EV isolation, detection, and analysis, addressing challenges posed by their nanoscale dimensions and complex biofluid matrices^[22,143]^. Advances in nanostructure-based isolation techniques and the development of high-sensitivity sensors have enabled more precise and efficient enrichment and characterization of EVs^[66,143,144]^. These technologies leverage physical and chemical properties such as size, surface charge, and affinity interactions to selectively capture and isolate EVs from complex biological samples. For instance, plasmonic, fluorescent, magnetic, and carbon-based nanomaterials have been widely used due to their exceptional sensitivity and specificity at the nanoscale, enabling enhanced molecular detection and bioinformatics profiling of EVs^[143,145]^. Furthermore, these nanomaterials facilitate single-EV analysis, revealing detailed molecular signatures and heterogeneity within EV populations, which is critical for biomarker discovery and therapeutic monitoring^[146,147]^. The integration of nanofabricated devices with advanced analytical tools enables more comprehensive characterization of EVs, thereby opening new avenues for biomedical research, particularly in liquid biopsy-based diagnostics and personalized medicine^[66,148]^. Ongoing research on EV biogenesis, molecular composition, and EV-targeting mechanisms is expected to advance the clinical translation of EVs in nanomedicine^[149]^. As research progresses, nanomaterial-based approaches continue to improve EV isolation efficiency and detection sensitivity, paving the way for clinical translation.

### Advanced nanotechnologies for EV isolation

Advanced nanotechnologies have been developed for the targeted and non-targeted isolation of EVs. Physical feature-based capture, lipid probes, affinitive molecules, and traditional centrifugation are essential for nontargeted isolation^[66]^. Targeted isolation uses specific molecules with a high chemical affinity for EVs, which simplifies EV-based liquid biopsy; however, their capture efficiency must be optimized. Therefore, a combination of nanostructures and chemical affinities offers enhanced efficiency and reduced complexity.

**Figure 1:**
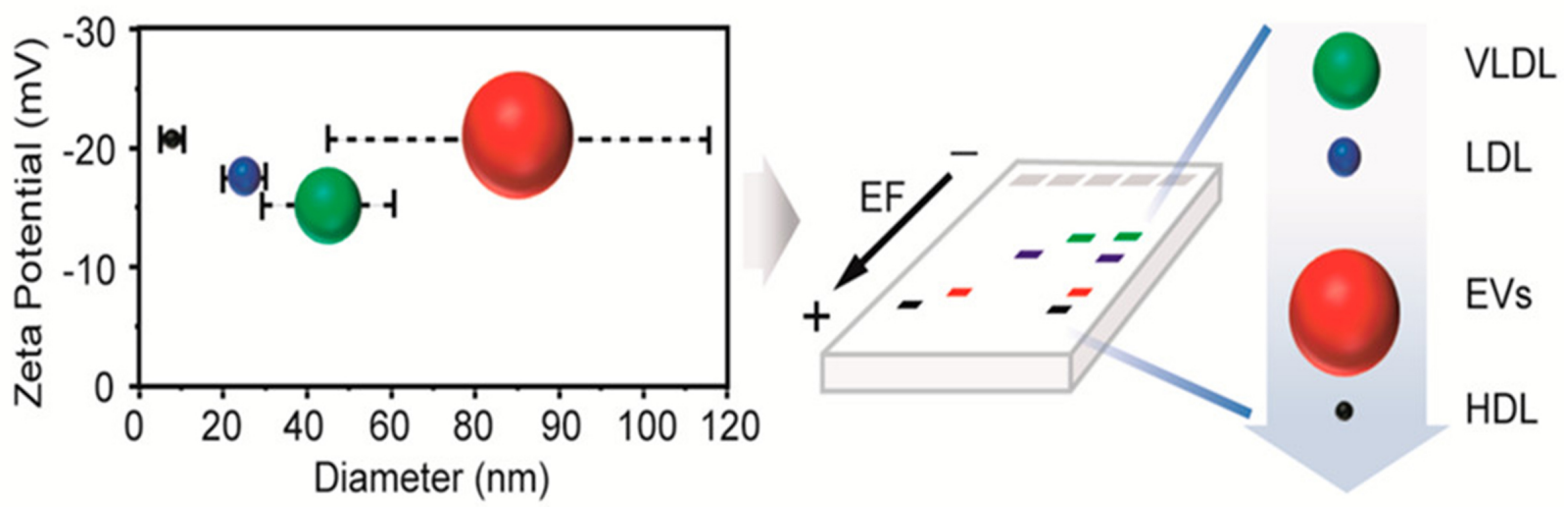
Schematic diagram of the sizes and zeta potentials of EVs and lipoproteins. The diagram illustrates the overlap between HDL, LDL, and VLDL in size and zeta potential with EVs, and the isolation of EVs from lipoproteins using agarose electrophoresis. Reproduced with permission from^[153]^. EVs: Extracellular vesicles; HDL: high-density lipoprotein; LDL: low-density lipoprotein; VLDL: very low-density lipoprotein; EF: electric field.

Physical property-based EV isolation relies on size distribution, leading to various isolation strategies^[150]^. Series filtration is the most common strategy; however, channel blockage due to impurity size and fluid shear forces hinders its widespread use^[151,152]^. Other size-based EV isolation procedures include electrophoresis and Marangoni flows. Electrophoresis shows different migration rates in an electric field, depending on the charge-to-size ratio [Figure 1]^[153]^. Marangoni flow, a contact-free method, generates a surface-tension gradient on the liquid surface, resulting in a recirculating flow^[154,155]^. EVs can also be separated based on their hydrodynamic characteristics using asymmetric flow field-flow fractionation^[156]^. Asymmetric flow field-flow fractionation can separate nanoscale soluble particles based on their density and hydrodynamic properties; however, the complicated fabrication and operation of these devices limit their application in biomedical research^[157,158]^. Deterministic lateral displacement is a traditional technique used to separate microscale particles based on pillar arrays. It has been used to isolate and separate EVs and integrate them into microfluidic platforms^[159,160]^. However, other EV characteristics such as density and stiffness can interfere with deterministic lateral displacement-based EV isolation^[161]^. Dielectrophoresis (DEP), an electrokinetic technique, enables NP transport in an asymmetric electric field. The DEP force scales with particle size and is governed by field frequency–dependent permittivity and conductivity, enabling selective separation of heterogeneous EV populations within microfluidic systems. For instance, Ibsen *et al.* developed a technique to isolate EVs using an alternating-current electrokinetic microarray chip with a DEP separation force^[162]^. In this system, nanoscale vesicles are attracted to high-field regions at microelectrode edges due to dielectric contrast with the surrounding biofluid, while larger cells migrate to low-field regions and small biomolecules remain largely unaffected, enabling efficient size- and property-dependent EV isolation. In 2019, Moore *et al.* integrated EV electrical conductance into a DEP-based microfluidic system to selectively enrich tumour-specific EVs, exploiting the higher conductance of tumour-derived vesicles arising from increased membrane fluidity associated with highly unsaturated lipid compositions compared with less invasive tumour cells^[163]^. However, exposure to non-uniform electric fields during DEP processing may alter EV membrane integrity, cargo composition, or functional activity, thereby potentially biasing downstream molecular and biological analyses.

**Figure 2:**
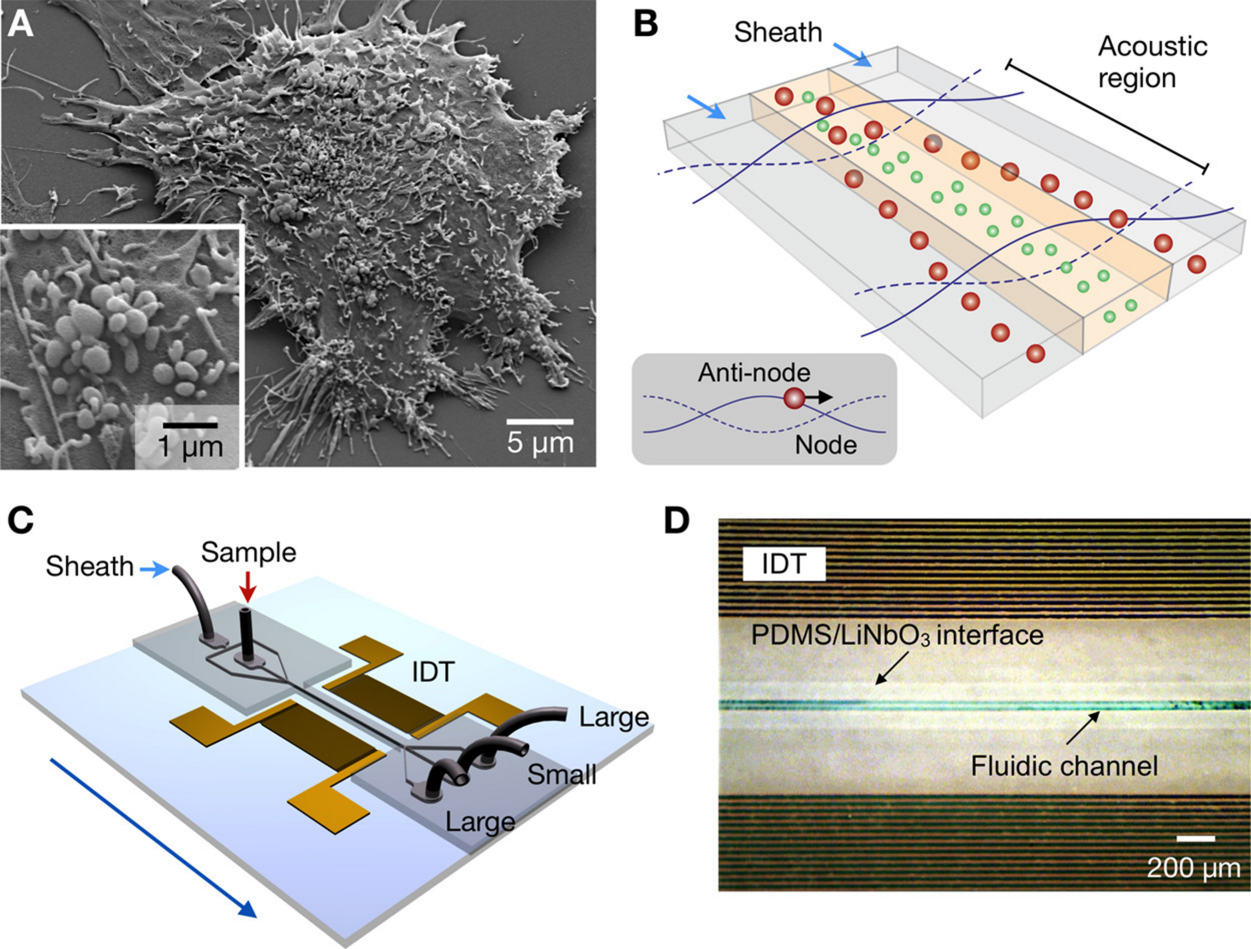
Acoustic nanofilter for MVs label separation. (A) Scanning electron microscopy image of MVs produced by human brain tumor cells (GBM20/3); (B) Schematic of filter operation. MVs in the acoustic region are moved to the nodes of the acoustic pressure region while under acoustic radiation (inset) pressure. As the acoustic force is related to MV volume, larger MVs travel faster and get eliminated by sheath flows positioned in the node region, whereas small MVs are retained by the center flow; (C) Schematic of the device. An acoustic wave is generated across the flow direction employing a pair of IDT electrodes. Small MVs are gathered at the center outlet, whereas large MVs are gathered at the two side outlets; (D) Micrographs showing a working prototype. A piezoelectric (LiNO_3_) substrate served as the pattern for IDT electrodes. The substrate and fluidic channel were irreversibly fused together. Reproduced with permission from^[165]^. MVs: Microvesicles; IDT: interdigitated transducer; PDMS: polydimethylsiloxane.

Surface acoustic waves are elastic mechanical waves that can be used to isolate EVs based on their mechanical properties. This method can be further modified to isolate EVs < 200 nm from whole blood achieving > 99.999% removal of blood cells [Figure 2]^[164,165]^. As illustrated in Figure 2, this separation strategy employs standing ultrasound waves to exert size- and density-dependent acoustic radiation forces on microvesicles (MVs). Larger MVs experience stronger acoustic forces and migrate more rapidly toward pressure nodes, while smaller vesicles remain closer to the center streamline. By optimizing the design of the interdigitated transducer (IDT) electrodes and associated electronics, high separation yield and resolution were achieved, with an electronically tunable size cut-off that enables *in situ* control of MV size selection. In this acoustic nanofilter, sheath flows positioned at the pressure nodes remove larger MVs through side outlets, whereas smaller vesicles are retained in the central outlet. The device, fabricated by patterning IDT electrodes on a piezoelectric LiNbO_3_ substrate and permanently bonding a microfluidic channel, was successfully applied to isolate nanoscale (< 200 nm) vesicles from cell culture media as well as MVs from stored red blood cell products. Surface acoustic waves-based acoustic nanofilters offer a contact-free and size-tunable method for EV isolation; however, they cannot separate farraginous cell debris and protein aggregates. Thermophoresis is a liquid-phase-based isolation method in which NPs migrate from high- to low-temperature regions due to collisions with solvent molecules at higher temperatures. This method has been used to profile surface biomarkers and detect miRNAs inside EVs^[166,167]^. Furthermore, thermophoresis-based EV-derived programmed cell death ligand 1 (PD-L1) detection provides suggestive data for early cancer diagnosis and immunotherapy response prediction^[168]^. Other liquid-phase-based detection methods, including artificial virus-mimicking fusogenic vesicles (Vir-FV) and droplet digital ExoELISA chips, can detect EVs in the liquid phase, providing a homogeneous basis for EV counting and biomarker studies, making this a promising approach for EV isolation^[169,170]^.

**Figure 3:**
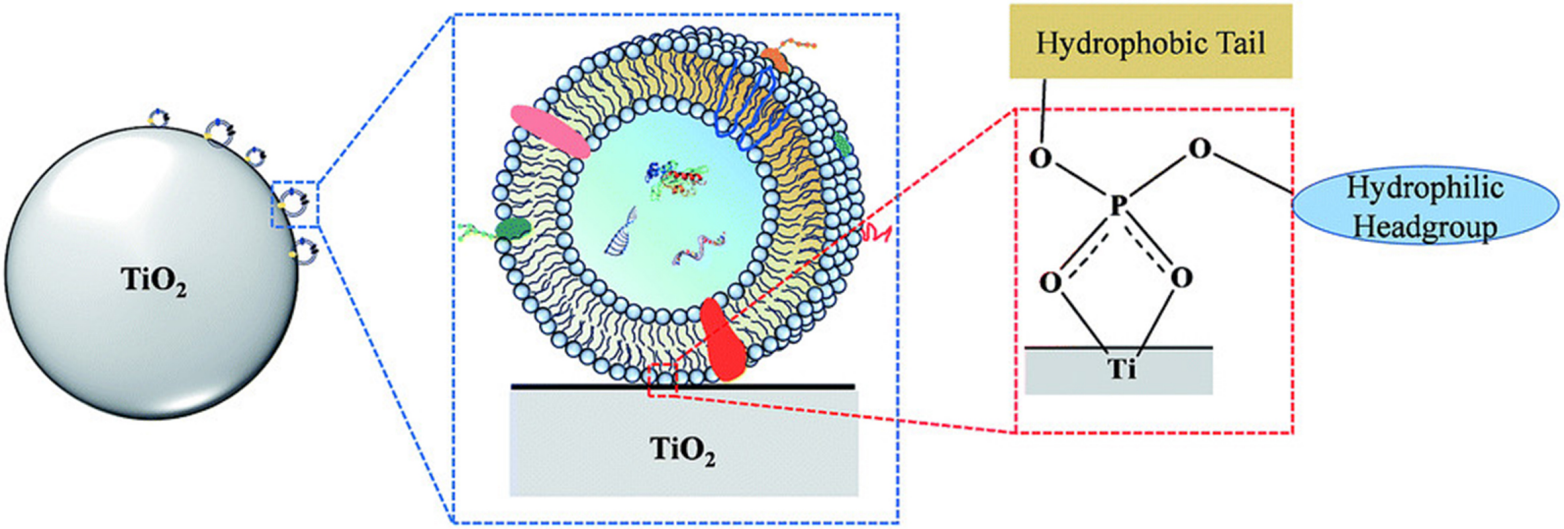
Mechanism for TiO_2_-based exosome isolation. TiO_2_ particles are utilized for exosome enrichment through bidentate binding between phosphate groups on the lipid-bilayer surface and TiO_2_. Reproduced with permission from^[177]^.

Chemical-affinity-based EV isolation can be divided into non-targeted and targeted capture methods. Nontargeted capture uses lipid probes, phosphatidylserine (PS) affinity molecules, and TiO_2_, whereas targeted capture uses antibodies, aptamers, and peptides. Lipid molecules on EV surfaces serve as crucial anchors for affinity-mediated capture. In 2017, Wan *et al.* designed a lipid nanoprobe system to label EVs for enrichment^[171]^. This system includes 1,2-distearoyl-*sn*glycero-3-phosphorylethanolamine, polyethylene glycol, and biotin, with an optimized cholesterol-polyethylene glycol 1,000-biotin probe^[171,172]^. This approach minimizes damage to EVs and can be integrated into functional assays. The outer membranes of EVs are enriched in cone-shaped lipids such as PS, which have been used for EV capture due to their affinity. Annexin A5 has been used for cell apoptosis detection and for improving EV isolation efficiency^[173,174]^. T-cell membrane protein 4 is a natural receptor for PS molecules, leading to the development of a T-cell membrane protein 4-based EV capture protocol^[175]^. Other PS-philic molecules, such as milk fat globule epidermal growth factor VIII (MFGE8), have also been explored in EV isolation studies^[176]^. Moreover, the hydrophilic surface of TiO_2_ can efficiently bind to phosphate groups on EV surfaces, making them potential targets for EV capture. In 2019, Gao *et al.* captured EVs from serum using TiO_2_ microspheres and Fe_3_O_4_@TiO_2_ NPs [Figure 3]^[177]^. Antibodies targeting EV biomarkers, including CD63, CD9, and CD81, and cancer-specific membrane proteins, such as epithelial cell adhesion molecule (EpCAM), epidermal growth factor receptor (EGFR), and glypican-1 (GPC-1), are popular tools for EV capture and isolation^[178-180]^. Additionally, aptamers, an alternative to antibodies for protein targeting, bind to antigens such as CD63, EpCAM, protein tyrosine kinase 7 (PTK7), prostate-specific membrane antigen (PSMA), platelet-derived growth factor (PDGF), and human epidermal growth factor receptor 2 (HER2). Owing to their lower dissociation constants and reduced risk of triggering undesirable immune responses compared with antibodies, aptamers have been utilized in EV isolation^[169,181,182]^. Similarly, peptides with specific affinity for proteins on the EV surface have been used for EV isolation, including EGFR ligands and tumor antigenic peptides for capturing major histocompatibility complex class I (MHC-I)+ EVs^[183]^.

Although chemical affinity-based EV isolation has significantly advanced EV-based liquid biopsy, significant challenges remain in making these techniques clinically viable. Incorporating nanostructures into EV isolation enhances reaction efficiency while addressing issues such as interfacial EV binding, microscale mass-transfer limitations, and boundary effects. Thus, the combination of nanostructures with chemical affinity represents a new paradigm for high-efficiency EV isolation. Thus, structural designs for micro- and nanoscale interfacial materials, such as nanowires, nanowire-on-micropillar arrays, porous structures, wrinkled structures, stacked nanospheres, and other on-demand structures, have been developed for EV capture. Graphene-based materials are used for wrinkled nanostructures^[184,185]^, whereas porous silicon nanowire-on-micropillar structures are used for effectively trapping EVs at interstitial sites between ciliated micropillars^[186]^. Similarly, a silicon nanowire-embedded polydimethylsiloxane substrate can be utilized for the electrostatic collection of urinary EVs. Nanointerfaces can be customized with nanostructured SiO_2_, and synthetic materials such as poly(amidoamine) dendrimers can provide sufficient steric hindrance to EVs^[182,187,188]^. In summary, various nanostructures have been developed for advanced EV capture nanotechnologies, enabling a high affinity for EVs and increased contact between EVs and the capture interface^[188-193]^.

### Advanced nanotechnologies for EV detection

Advanced sensors and single-EV detection have reduced the limit of detection (LOD) of EVs, addressing their heterogeneity and the urgent demand for sensitive detection technologies. Optical sensors that detect fluorescent and spectral signals are used for EV detection. Fluorescent signals are detected by linking probes to antibodies or aptamers^[66]^. Different modes of fluorescent signal generation have been adopted to improve the LODs of EVs. Spectral signal sensing techniques, including near-infrared (NIR) spectroscopy, surface plasmon resonance (SPR), and surface-enhanced Raman spectroscopy (SERS), have also been used. Fluorescence quenching is a more sensitive method than direct imaging for EV detection. Graphene oxide (GO) is the first 2D material identified with fluorescence quenching ability, as it binds to aptamers on its surface through strong π–π stacking interactions, where fluorescence is quenched via fluorescence resonance energy transfer between dyes and GO^[181]^. This allows EVs with specific surface proteins (e.g., miR-193a) to compete and display fluorescent signals^[194]^. Fluorometric nanosensors have also been developed for this purpose using MoS_2_-incorporated multi-walled carbon nanotubes (MWCNTs), quenching phycoerythrin-conjugated CD63 antibodies, achieving an LOD of 1,480 EVs/μL^[195]^. Further, nucleic acid base pairing can also control fluorescent signals. A molecular beacon, a hairpin-shaped ssDNA with a quenched fluorophore, can restore fluorescence upon binding to a complementary sequence, such as an EV-derived miRNA^[196]^. Notably, researchers have designed molecular beacons for the simultaneous detection of miRNAs and surface proteins, such as Vir-FV^[177,196]^.

**Figure 4:**
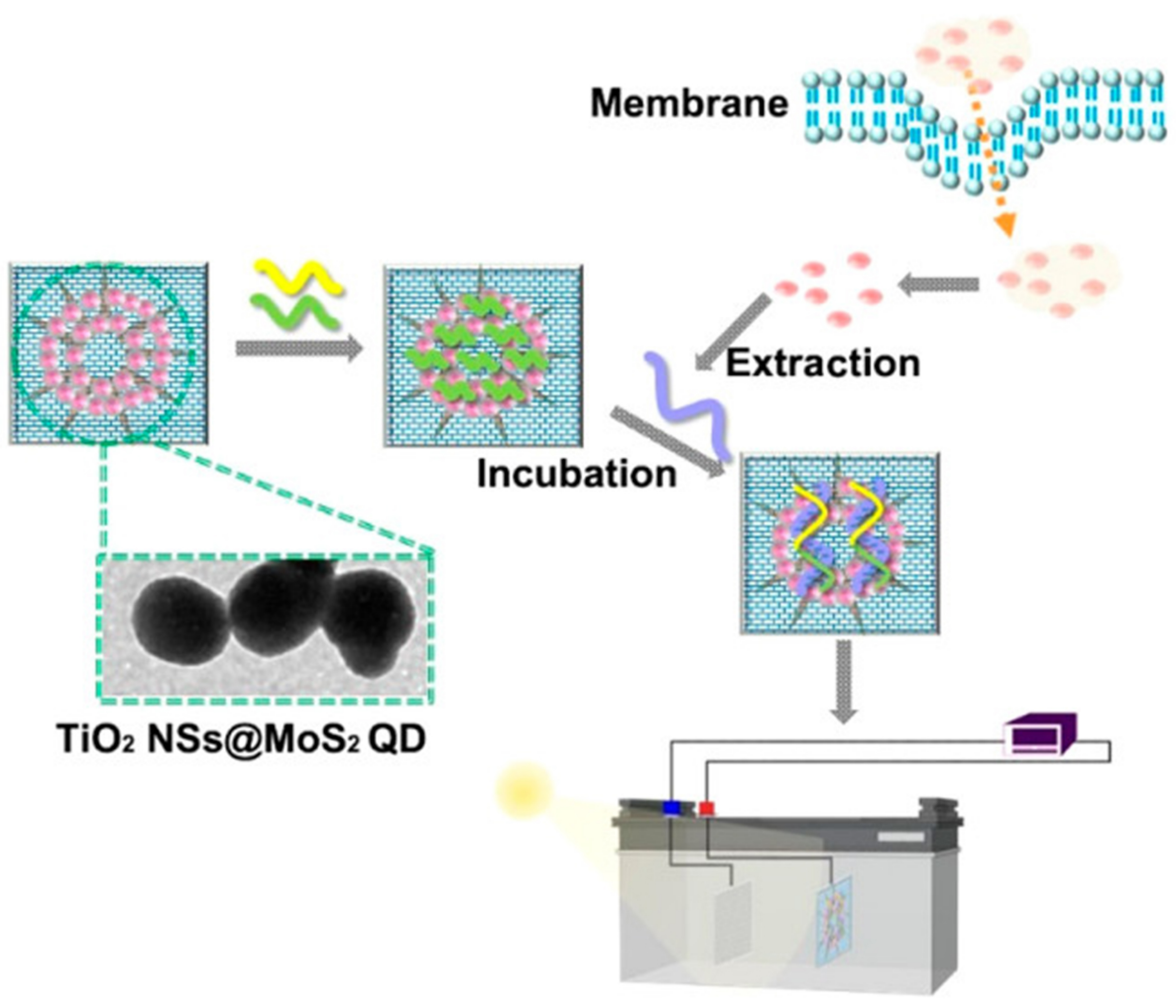
Schematic of a self-powered biosensing device based on TiO_2_ NSs@MoS_2_ QDs for the quantitative detection of exosomal RNA (Homo sapiens HOXA distal transcript antisense RNA, HOTTIP). Reproduced with permission from^[199]^. NSs: Nanostructures; QDs: quantum dots.

**Figure 5:**
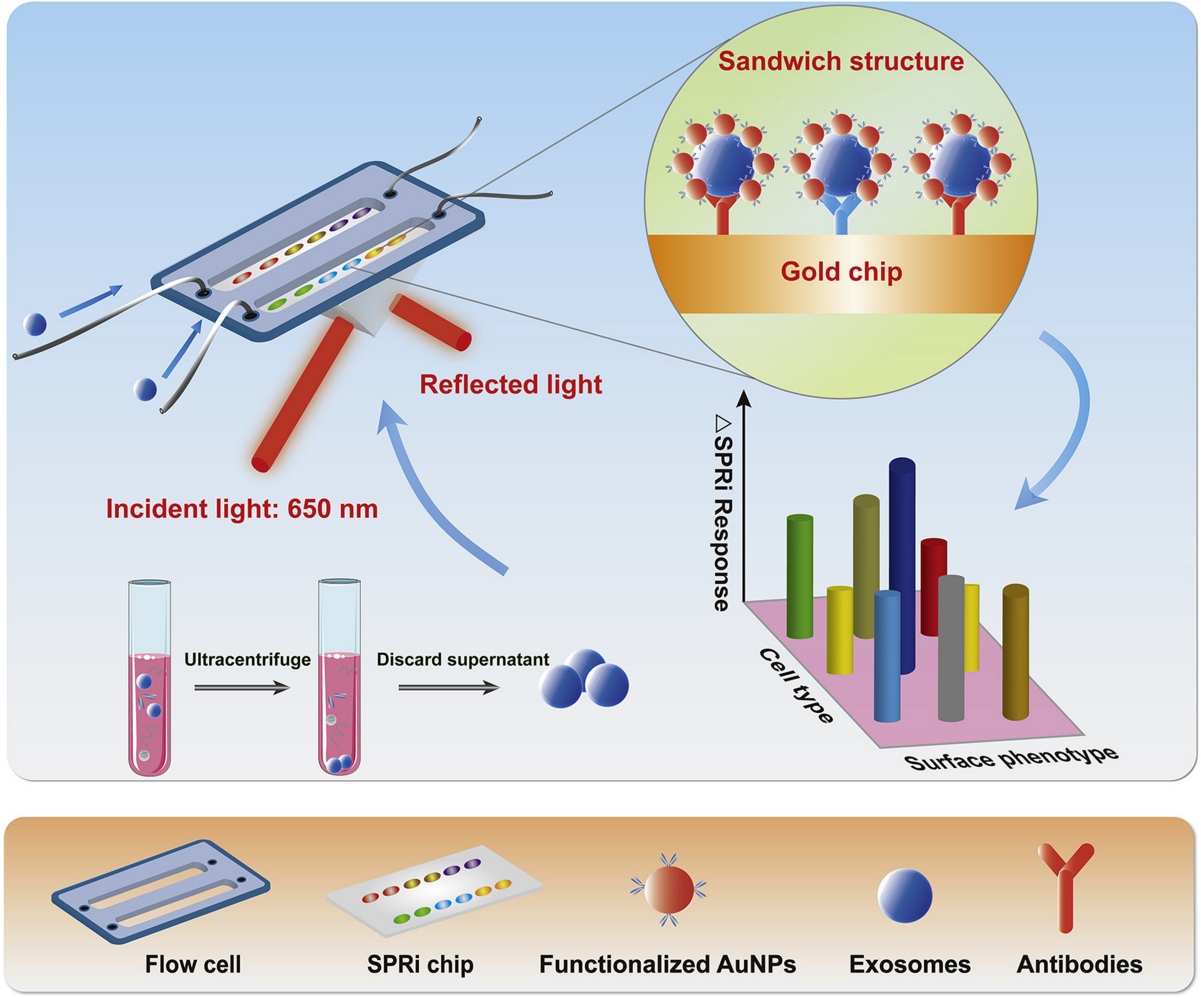
Schematic of the SPRi biosensor developed for the high-sensitivity and multiplex characterization of non-small cell lung cancer-derived exosomes through bioaffinity interactions of antibodies and various recognition sites. Reproduced with permission from^[201]^. SPRi: Surface plasmon resonance imaging; AuNPs: gold nanoparticles.

Metal-enhanced fluorescence is another optical sensing method for detecting EVs. Metal-enhanced fluorescence uses an antibody-conjugated magnetoplasmonic nanorod to capture EVs, enabling the release of miRNAs and their detection by molecular beacons that bind to the nanorod^[197]^. This fluorescence signal can be further amplified using Au plasmons. Similarly, quantum dots (QDs) have unique optical properties, including broad, intense absorption and flexible excitation, which enable higher fluorescence quantum yields. They have been used to analyze EV-derived miRNAs and detect pancreatic cancer-associated EV biomarkers such as EpCAM and Ephrin type-A receptor 2 (EphA2)^[198]^. The Self-powered TiO_2_@MoS_2_ QD-based probe shows ultrasensitive detection of EV-derived HOXA transcript at the distal tip (HOTTIP) RNA, achieving a detection limit of 5 fg/mL [Figure 4]^[199]^. QDs can easily be enhanced, and their combination with photonic crystals can lead to diagnostic biochips for EV detection^[200]^. Because spectral signals offer lower background and better LOD than fluorescence, this property can be used to accurately identify circulating EVs for cancer diagnosis. For example, Lyu *et al.* synthesized a NIR afterglow luminescent nanosensor by incorporating quencher-tagged aptamers into a NIR-semiconducting polyelectrolyte complex. When the targeted EVs bind to the aptamers, the distance between the polyelectrolyte complex and quencher increases, activating the afterglow signal^[200]^. SPR signal detection is another sensitive label-free technology that detects molecular interactions on Ag/Au surfaces and can be used for multiplex detection of EVs derived from lung cancer cells[Figure 5]^[201]^. This design can be further modified to achieve higher sensitivity using dual-gold NPs (AuNPs) or Au nanocluster membrane–EV–Au nanorod complexes^[202,203]^.

Electrochemical sensors represent another advanced nanotechnological approach to EV detection. Horseradish peroxidase (HRP)-based colorimetry and electrochemical luminescence are widely used for EV detection on a 3,3′′,5,5′′-tetramethylbenzidine substrate^[204]^. Ultrasensitive nanoprobes, such as ferrocene, Ti_3_C_2_ MXene, and metal ions, can improve the performance. Dual-signal detection systems such as nanointerdigitated electrodes, CG-quadruplex/hemin, and aptamer-conjugated Ti_3_C_2_ MXenes have been explored for EV detection owing to their large surface areas and catalytic activities^[205]^. Notably, an enhanced LOD of 30 EVs/μL was achieved using AuNP-enhanced Ti_3_C_2_ MXenes for EV detection^[206]^. Moreover, a methylene blue-labelled aptamer has been used in ultrasensitive HRP nanoprobes for EV detection, with an LOD of 0.1 EVs/μL achieved by doping ferrocene onto a ZIF-67/ITO film and immobilizing black phosphorus nanosheets^[207]^. Similarly, a range of metal ions with HRP activity, such as Cu^2+^, Fe^3+^, and Ru(bpy)_3_^2+^ (bpy = 2,2‘-bipyridine), have been used as electrochemical sensors for EV detection. Techniques include antibody-conjugated CuS-enclosed microgels^[208]^, aptamer-capped Fe_3_O_4_ NPs^[209]^, and Au-loaded Fe_3_O_4_ nanocubes^[210]^. Moreover, the hemin/G-quadruplex system is a popular nanosensor for EV detection, with high selectivity and sensitivity for cancer-derived EVs, and it analyzes the DNAzyme and NADH activities of EVs. Xu *et al.* developed an on-chip hemin/G-quadruplex system with a LOD of 4.39 EVs/μL. For instance, a two-stage microfluidic platform (ExoPCD-chip) integrating on-chip exosome isolation with *in situ* electrochemical analysis was developed using a CD63 aptamer and a mimicking DNAzyme strategy. Binding of CD63-positive exosomes opens a hairpin DNA probe, releasing a G-rich sequence that forms a hemin/G-quadruplex DNAzyme, enabling continuous H_2_O_2_ generation and strong signal amplification. Enhanced capture was achieved via staggered Y-shaped micropillars and phosphatidylserine–Tim4–based magnetic enrichment, allowing detection of CD63-positive exosomes down to 4.39 × 10^3^ particles mL^-1^ over a five-order linear range^[175]^. Huang *et al.* modified the above system by adding a dual-signal amplification scheme with linker-DNA amplification, achieving a LOD of 0.954 EVs/μL^[211]^.

In addition to optical and electrochemical sensors, several other sensors can be used to analyze EVs in body fluids, including field-effect transistors (FET) and magnetic and mechanical sensors. Electric fields regulate conductivity and transform biological signals into electrical signals, making FET sensors highly sensitive. Two such examples are an AuNP-decorated reduced GO FET nanosensor for label-free quantification of plasma EVs and a reduced GO-based FET biosensor for EV detection with a LOD of 20 EVs/μL^[212,213]^. FET sensors can also be used to detect EVs in miRNAs^[214]^. Magnetic sensors can differentiate between healthy individuals and patients with ovarian cancer. One such example is the 2D magnetic MoS_2_-Fe_3_O_4_ hybrid nanostructure^[215]^. Similarly, electrokinetic and mechanical sensors have demonstrated promising results for EV detection, offering potential alternatives with unique advantages in terms of sensitivity and specificity^[216,217]^.

The heterogeneity of EVs presents significant challenges for their analysis in body fluids. High-resolution techniques, such as DGC, have revealed a more confined range of molecules within EVs and indicated that proteins in EVs of different sizes exhibit distinct glycosylation and phosphorylation levels^[40,218]^. Single-cell EV analysis has provided insights into the characteristics of EVs from specific cells; however, traditional population measurements still obscure the diversity of EVs secreted by the same cell, underscoring the need for single-EV detection. Techniques such as total internal reflection fluorescence microscopy, DNA point accumulation for imaging in nanoscale topography, and advanced platforms such as ExoELISA chips have been developed to enable single EV detection^[169,219,220]^. However, multiplex profiling at the single EV level remains a significant challenge. Innovations such as NGS-based platforms and microbead-assisted flow cytometry have opened new possibilities^[221-223]^. Although capturing detailed information from individual EVs remains challenging, continued advancements in analytical tools hold promise for future breakthroughs in single EV detection.

## NANOTECHNOLOGIES IN cfDNA RESEARCH

Despite the availability of commercially developed kits for cfDNA analysis, their detection efficiencies and accuracies are often limited. These limitations arise from time-consuming, complex enzymatic amplification processes or from detection limits that are too close to clinically relevant quantities, reducing their overall effectiveness in practical applications^[224]^. Moreover, given the broad physiological concentration range of cfDNA, which varies from low (ng/mL) to high (µg/mL) depending on the cancer type, clinically accurate detection is often challenging^[225]^. Therefore, a good sensor must be able to detect as wide a concentration range as possible.

**Figure 6:**
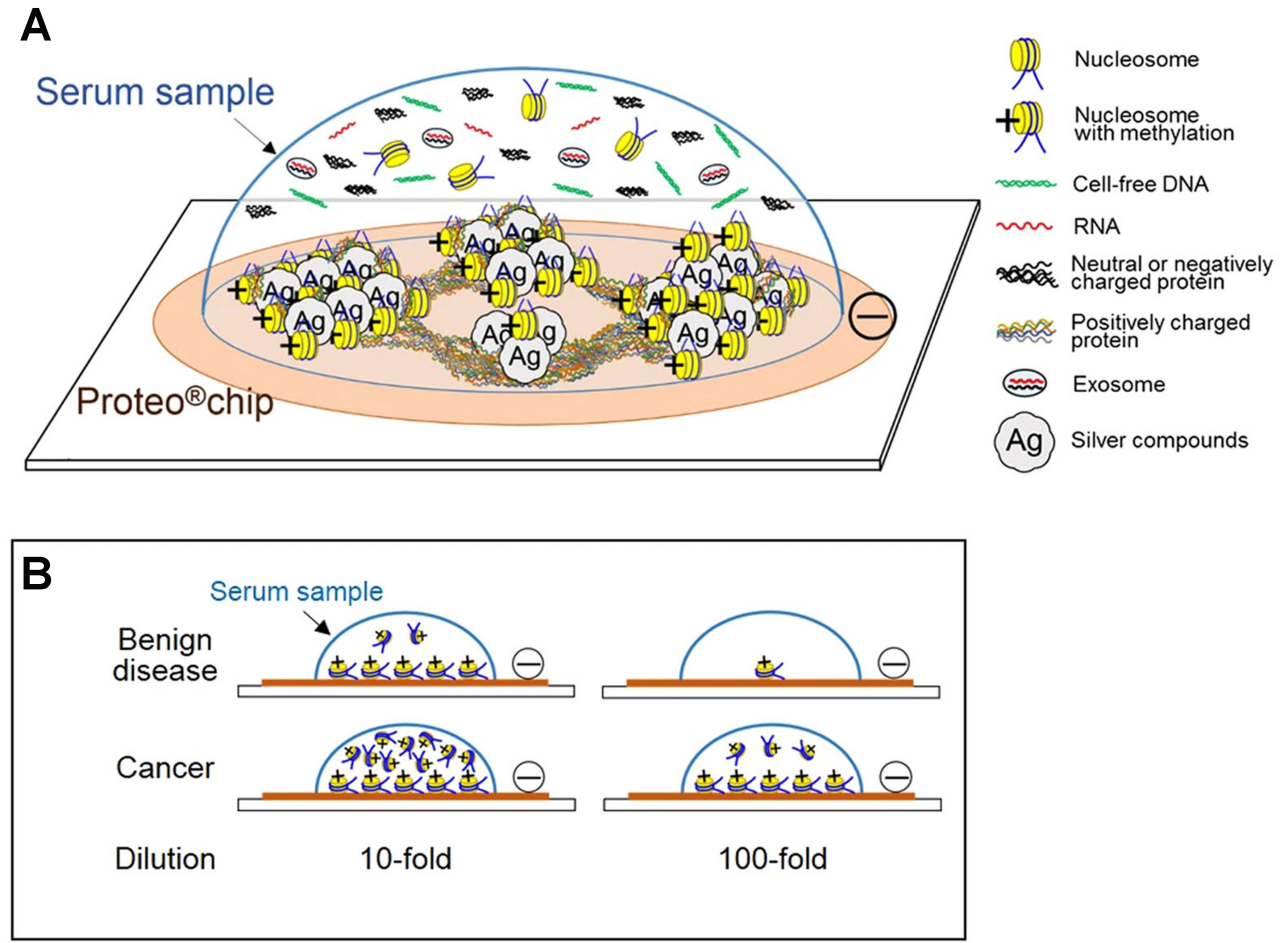
Serum components interacting with a silver nanoscale hexagonal column chip. The surface of the Proteo®chip has a negative charge, which is designed to capture positively charged histones from nucleosomes circulating in the blood. The key is that when the DNA in these nucleosomes is methylated, it causes the histones to keep their positive charge. As a result, the nucleosomes that carry these methylation markers bind readily to the chip’s surface (A). This allows clear discrimination between serum samples from patients with cancer and those from patients with benign disease, even when the samples are diluted (B). Reproduced with permission from^[228]^.

To address these issues, detection techniques must be capable of analyzing cfDNA across a broad concentration spectrum, ensuring accuracy and reliability in diverse clinical scenarios in a timely manner. One such example is the magnetic NP-based inverse sensitivity response assay^[226]^. This exploits the electrostatic interaction between hexadecyltrimethylammonium bromide-coated Au nanorods and DNA, leading to unique, DNA-concentration-dependent particle aggregation^[226,227]^. This colorimetric assay exhibits inverse sensitivity: lower analyte concentrations yield greater sensing responses. It effectively covers a wide range of cfDNA concentrations, from 20 ng/mL to 10 µg/mL, and can analyze various cancer types^[226]^. Another example of detecting cfDNA is the utilization of characteristic peaks of SERS spectra^[228,229]^. For instance, Ag nanoscale column chips can be used to distinguish individuals with gastric and colorectal cancer from healthy individuals and patients with benign disease by analyzing discrete SERS peaks [Figure 6]^[228]^.

**Figure 7:**
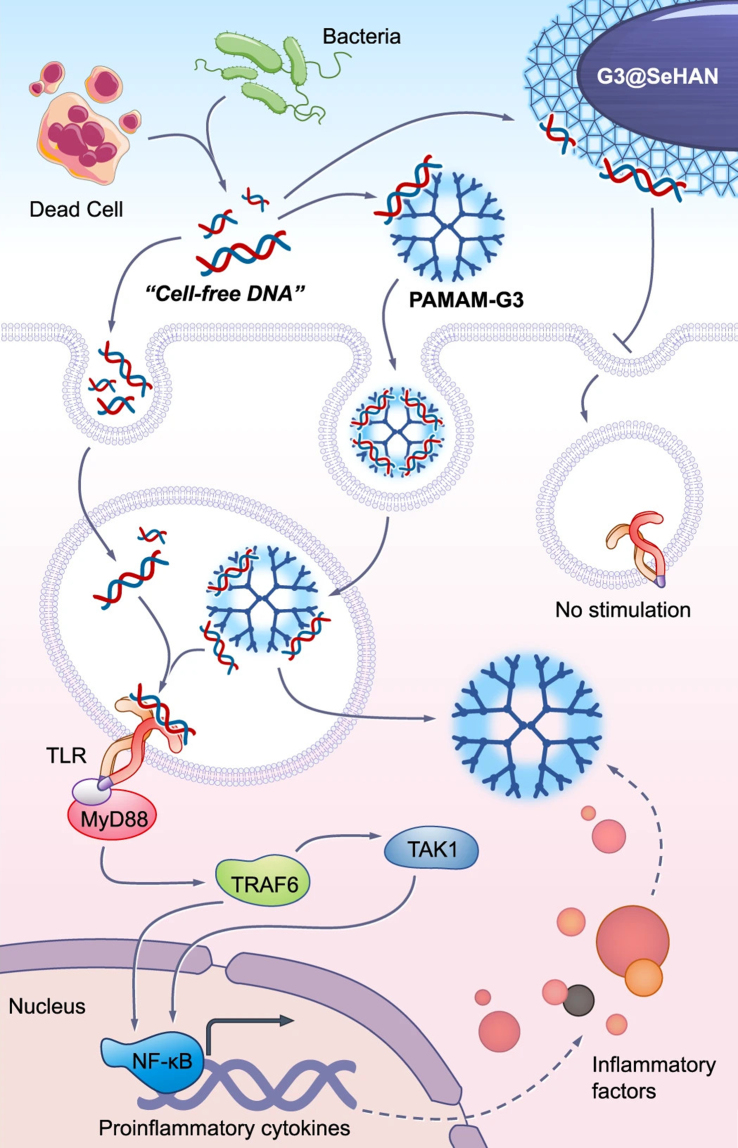
cfDNA-scavenging mechanisms of G3@SeHANs and PAMAM-G3 for the detection of periodontitis. cfDNA released from dead cells or bacteria is present in the extracellular environment and can be internalized by immune cells through endocytosis. Free cfDNA can traffic to endosomal compartments, where it engages TLRs, leading to recruitment of MyD88 and downstream activation of TRAF6 and TAK1. This signaling cascade culminates in nuclear translocation of NF-κB and the induction of pro-inflammatory cytokines and inflammatory mediators. PAMAM-G3 binds cfDNA and is internalized into endosomes; however, the cfDNA–PAMAM-G3 complexes remain capable of stimulating endosomal TLR signaling, thereby sustaining inflammatory responses. In contrast, G3@SeHANs efficiently scavenge cfDNA in the extracellular space and prevent its productive interaction with endosomal TLRs following cellular uptake. By sequestering cfDNA and blocking TLR–MyD88 signaling, G3@SeHANs suppress downstream activation of TRAF6, TAK1, and NF-κB, resulting in reduced transcription of pro-inflammatory cytokines and attenuation of inflammatory factor release. Reproduced with permission from^[230]^. cfDNA: Cell-free DNA; G3@SeHANs: generation 3 selenium–heparin-based anionic nanostructures; PAMAM-G3: poly(amidoamine) generation 3 dendrimers; TLRs: Toll-like receptors; MyD88: myeloid differentiation primary response protein 88; TRAF6: tumor necrosis factor receptor–associated factor 6; TAK1: transforming growth factor beta–activated kinase 1; NF-κB: nuclear factor kappa B.

Another research group developed a nanoparticulate cfDNA scavenger tailored for periodontitis by coating selenium-doped hydroxyapatite nanoparticles (SeHANs) with cationic polyamidoamine dendrimers [poly(amidoamine) generation 3 dendrimers (PAMAM-G3)] to create G3@SeHANs^[230]^. When analyzing the activities, both G3@SeHANs and soluble PAMAM-G3 polymer were effective at scavenging cfDNA, reducing periodontitis-related inflammation *in vitro*, and mitigating inflammatory bone loss in a mouse model of ligature-induced periodontitis [Figure 7]. Further, G3@SeHANs modulated the mononuclear phagocyte system, favoring the anti-inflammatory M2 macrophage phenotype over the proinflammatory M1 phenotype. *In vivo*, G3@SeHANs outperformed PAMAM-G3 in decreasing inflammation and alveolar bone loss. These findings emphasize the critical role of cfDNA in periodontitis and suggest that hydroxyapatite-based nanoparticulate cfDNA scavengers may be a promising therapeutic strategy for managing this disease.

## CONCLUSIONS

In summary, EV-DNA and ccfDNA are emerging as key biomolecules for minimally invasive approaches to ovarian cancer detection and monitoring. Current studies have highlighted that EV-DNA comprises a richly heterogeneous genetic material of nuclear and mitochondrial origin, reflecting the complex genomic structure of primary tumors. Simultaneously, high diagnostic and prognostic values, especially mutation and methylation profiles, have already been determined using ccfDNA analyses.

However, significant challenges remain, including methodological variability, low analyte abundance, and the need for standardized protocols. Improvements in long-read sequencing and nanotechnology-based isolation and detection methods will help overcome these limitations and facilitate the clinical translation of EV-DNA and ccfDNA as biomarkers. Further research on the biological underpinnings and functional roles of these DNA fragments will enable their integration into multi-omics approaches, potentially offering unparalleled insights into tumor biology and treatment resistance. The continued development and clinical validation of EV-DNA and ccfDNA assays are essential for their translation into routine oncological practice, enabling earlier detection, personalized therapies, and improved outcomes for patients with ovarian cancer.

## DECLARATIONS

### Copyright

© The Author(s) 2026.
